# Eurythmy Therapy in clinical studies: a systematic literature review

**DOI:** 10.1186/1472-6882-8-8

**Published:** 2008-03-31

**Authors:** Arndt Büssing, Thomas Ostermann, Magdalena Majorek, Peter F Matthiessen

**Affiliations:** 1 Chair of Medical Theory and Complementary Medicine, University of Witten/Herdecke, Gerhard-Kienle-Weg 4, 58239 Herdecke, Germany; 2 Therapeutic Practice, Talstrasse 386, 4204 Himmelried, Switzerland

## Abstract

**Background:**

We aimed to overview the current literature on eurythmy therapy (EYT) which is an integral part of Anthroposophic Medicine. EYT can be described as a movement therapy in which speech movements are transposed into exercises which address the patient's capability to soul expression and strengthen his salutogenetic resources.

**Methods:**

We searched several databases such as Cochrane, EMBASE, NCCAM, NLM, DIMDI, CAMbase, and Medline for case-control studies, cohort studies and randomised controlled trials on the treatment effects of EYT in a clinical setting. In a second search we included journal databases from Karger, Kluwer, Springer, Thieme, and Merkurstab archive.

**Results:**

We found 8 citations which met the inclusion criterion: 4 publications referring to a prospective cohort study without control group (the AMOS study), and 4 articles referring to 2 explorative pre-post studies without control group, 1 prospective, non-randomized comparative study, and 1 descriptive study with a control group. The methodological quality of studies ranged in from poor to good, and in sample size from 5 to 898 patients. In most studies, EYT was used as an add-on, not as a mono-therapy. The studies described positive treatment effects with clinically relevant effect sizes in most cases.

**Conclusion:**

Indications, study designs and the usage of additional treatments within the identified studies were quite heterogeneous. Despite of this, EYT can be regarded as a potentially relevant add-on in a therapeutic concept, although its specific relevance remains to be clarified. Well performed controlled studies on this unique treatment are highly recommended.

## Background

Complementary and alternative medicine (CAM) has become increasingly popular over the last decades. According to Bausell et al. [1], especially patients with chronic diseases increasingly seek for CAM-therapies. With a growing amount of health information in the internet, physicians and therapists and patients are often not prepared to judge provided information of CAM-health care approaches properly. Information dissemination of published evidence about the effectiveness of remedies and therapies therefore forms a neccesary basis for shared-decision making for patients and practitioners. While scientific evidence exists for some popular CAM therapies like acupuncture [2,3], well-designed scientific studies are lacking for most other CAM therapies.

One popular representative of the so called "Whole Medical Systems" with a complete system of theory and practice with a distinct usage of mind-body based approaches is Anthroposophic Medicine (AM). It was founded in the 1920s by the Austrian philosopher Rudolf Steiner and the Dutch physician Ita Wegman [4], and can be regarded as a complete medical system of theory and practice, with a distinct usage of mind-body based practices. The core concepts are that the body is not an independent material organism, and that health depends on a harmonious relationship between the physical body, vital force, soul, and ego. AM therefore intends to address the salutogenetic capacities of the patients, and to strengthen their autonomy [5]. For each patient, AM oriented physicians and therapists develop individual therapeutic strategies which – apart from conventional treatments – include Art Therapy (i.e. painting, drawing, clay modelling, music or speech exercises), Rhythmical Massage, Eurythmy Therapy (EYT), Counselling, and AM medication of mineral, botanical or zoological origin (mostly used in homeopathic dilutions). Because of this unique and individualized approach, only a few studies within this framework follow the conventional methodological conventions.

Much is known about the AM-based application of mistletoe extract in cancer patients [6-8], and a recent Health Technology Assessment by Kienle et al. [9] reviewed the effectiveness, benefit, efficiency and safety of AM. Because of the outstanding importance in AM based treatment of patients and some impressive (anecdotic) case reports, we intended to overview the current literature on the clinical effects of EYT.

EYT ("harmonious rhythm"), introduced by Rudolf Steiner in 1911, can be described as an active exercise therapy, involving cognitive, emotional and volitional elements [10]. Although there are some similarities with (psychotherapeutic) dance therapy, EYT is a unique movement therapy in which speech movements are transposed into exercises which address the patient's capability to soul expression and strengthen his salutogenetic resources. These specific body movements go along with meditative aspects in terms of guided imagery. EYT is assumed to have general effects (i.e. improving breathing patterns and posture, strengthening muscle tone, enhancing physical vitality [5]), and specific therapeutic effects. Moreover, EYT claims to stimulate somatic healing processes through the soulful experience of the respective movements [11].

## Methods

We intended to search for English or German language cohort studies and controlled trials which address the specific treatment effects of EYT in a clinical setting (inclusion criterion). Comments, opinions, and theoretical considerations were excluded. As broad search terms we used the most global term "eurythmy", and its German translation "Eurythmie" and its modification "Heileurythmie".

To get a first overview, the following electronical databases were used to find articles: MEDLINE, EMBASE, The Cochrane database, DIMDI and CAMbase. Finally, we also screened the journal databases of Karger, Kluwer, Springer, Thieme and the Merkurstab archive to find relevant information. In addition, an internet search was performed using Google Scholar adding the search terms "study" to the above search terms.

The complete search was performed in June and October 2007.

All articles found this way were fully read and their reference lists were checked for further relevant publications. Moreover, we contacted experts in the field of AM to find other relevant studies.

We graded the methodological quality of the studies by the following checklist: Adequate description of the subject assembly process (methods for patient selection described, eligible but not enrolled subjects and reason for exclusion), equality of comparison groups in the case of controlled studies, adequate description of subject follow up, adequate description of treatment, unbiased surveillance for adverse outcomes. The quality details of the relevant studies are discussed in the description of studies.

The reporting of the results adhered, if possible and appropriate, to the MOOSE guidelines [12].

However, as the studies were quite heterogeneous including a variety of perspectives, but not any randomised controlled trial, we decided to classify them roughly at first glance with respect to indication, treatment setting, research design, the number of patients involved and the outcome measures/results given in the studies/reports.

Where possible, we calculated an effect size (Cohen's d) or indicated at least Standardised Response Means (= mean change score divided by the standard deviation of the change score). According to Cohen [13] and Wolff [14], we judged effect sizes > 0.8 as indicators of large effects, and 0.5–0.8 for moderate effects. We judged effect sizes > 1.0 as relevant, which – according to Cohen [13] – indicate a non-overlap of > 55% in the two distributions.

## Results

### Search results

The DIMDI database revealed 18 hits for the term "eurythmy": 8 were duplicates, and 10 were identified as independent contributions (Table 1). However, some references had to be excluded because the term eurythmy was used in an another context, one was a German language interview on the students of Waldorf schools, and one was a German language comment by Ernst [15] on a clinical study by Majorek et al. [10]. One paper of Hamre et al. [16] focused on health costs in anthroposophic therapy users, and a study by Heusser et al. [17] addressed treatment pattern and compliance with AM, and thus both were excluded too.

**Table 1:** Citations of the term eurythmy found in the databases and their source

**Found in the first run of the search:**

1) Majorek *et al*., 2004: Therapeutic Eurythmy-movement therapy for children with attention deficit hyperactivity disorder (ADHD): a pilot study. MEDLINE
2) Hamre *et al*., 2004: Anthroposophic therapies in chronic disease: the Anthroposophic Medicine Outcomes Study (AMOS). MEDLINE
3) Hamre *et al*., 2006a: Anthroposophic therapy for chronic depression: a four-year prospective cohort study. MEDLINE
4) Hamre *et al*., 2007a: Eurythmy therapy in chronic disease: a four-year prospective cohort study. MEDLINE
5) Hamre *et al*. 2007b: Anthroposophic medical therapy in chronic disease: a four-year prospective cohort study. MEDLINE
6) Hamre *et al*., 2007c: Anthroposophic vs. conventional therapy for chronic low back pain: A prospective comparative study. EMBASE
* Ernst, 2004: Eurythmie beruhigt den Zappelphilipp. MEDLINE
* Hamre *et al*., 2006b: Health costs in anthroposophic therapy users: a two-year prospective cohort study. MEDLINE
* Heusser *et al*., 2006: Palliative in-patient cancer treatment in an anthroposophic hospital: I. Treatment patterns and compliance with anthroposophic medicine. MEDLINE
* Interview: Waldorfschulen – Selbstbewusst ins Leben, Deutsches Ärzteblatt
* Characteristics of lumbar segment injury of spinal cord in eurythmics athletes. EMBASE

**Found in the Second run of the search:**
7) Fischer K, Rheingans, 1985: Vergleichende Untersuchung einer künstlerisch-übenden mit einer konventionell aktiv-trainierenden Kurbehandlung an Herz- und Kreislaufkranken mit einer Herzinfarktgruppe. ERFAHRUNGSHEILKUNDE
8) Bräuner-Gülow and Gülow, 2006: Heileurythmie bei Magersucht im Jugendalter. Methodik zur Bewegungsanalyse. Aspekte zur Diagnostik, Bewegungstherapie und Forschungsstand. Zusammenfassung einer Pilotstudie von 2002–2005. Der Merkurstab

* not in accordance with criteria

Because the founding papers on eurythmy were in German language, we sought German language articles by performing a search in the database CAMbase and the archive of the journal "Der Merkurstab" (from 1946 to 2007). Within the "Merkurstab" archive we found 92 contributions referring to the term "Heileurythmie", while the adjective "heileurythmisch" appeared in 14 references. Most were theoretical reflections, announcements or reports of courses. Nevertheless, in this second run we found several German language case-reports (most of them were anecdotic if anything, or had a focus on the description of exercises rather than on the clinical outcome) and 2 additional studies. Because one can not exclude a publication bias among the case reports, which predominantly described beneficial effects, and only a few no significant clinical changes at all, they could be evaluated in a separate paper.

Although we found some other articles mentioning the usage of EYT within in a complex therapeutic setting (i.e. [17-22]), they were not included in this review, because our main intention was to focus on clinical trials which addressed the specific effects of EYT. Nevertheless, we diverged from this inclusion criterion only in case of the Anthroposophic Medicine Outcome Study (AMOS), which was published with respect to different details [23-26], but has to be regarded as one study, and described the number of patients with EYT usage.

### Description of studies

The search strategy identified 8 reports which met the inclusion criterion. With respect to the design, we had 4 publications referring to a prospective cohort study without control group (the AMOS study, which investigated the effectiveness and costs of AM therapies in outpatients with chronic disease), and 4 articles referring to 2 explorative pre-post studies without control group, 1 prospective, non-randomized comparative study, and 1 descriptive study with a control group (Table 2).

**Table 2:** Design of clinical studies enrolling therapeutic eurythmy (with and without add-on treatments)

**Reference**	**Indication**	**Research design**	**Number of patients**	**Durationof study**
**Fischer and Rheingans, 1985**	heart attack	prospective study **with control group**	39 (23 + 16) patients in a health resort	7 month
**Majorek *et al*., 2004**	attention deficit hyperactive disorder (ADHD)	exploratory pre-post case study without control group	5 boys (8–10 years)	9 month
**Bräuner-Gülow and Gülow, 2006**	anorexia nervosa	exploratory pre-post study without control group	70 girls/women (10–19 years)	4 years
Hamre *et al*., 2004	various chronic diseases (mental, respiratory, musculoskeletal, etc.)	prospective cohort study (pre-post design without control group (AMOS)	898 outpatients	4 years
Hamre *et al*., 2006a	depression	prospective cohort study (pre-post design) without control group (AMOS)	97 outpatients	4 years
**Hamre *et al*., 2007a**	various chronic diseases (mental, respiratory, musculoskeletal, headache etc.)	prospective cohort study (pre-post design without control group (AMOS)	419 outpatients	4 years
Hamre *et al*., 2007b	various chronic diseases (mental, respiratory, musculoskeletal, headache etc.)	prospective cohort study (pre-post design without control group (AMOS)	233 outpatients	4 years
**Hamre et al., 2007c**	Chronic low back pain	prospective non-randomized **comparative study **(AMOS)	62 (34 + 28) patients	1 year

The methodological quality of studies ranged from poor to good, and in sample size from 5 to 898 patients. The study of Hamre and co-workers [23-26] analysed patients with various chronic diseases (i.e. mental, respiratory, musculoskeletal, etc.), while the others investigated patients with heart attack, anorexia nervosa, depression, chronic low back pain, and attention deficit hyperactive disorder (ADHD), respectively.

In the identified pre-post studies, the description of enrolled patients and their diagnoses was fulfilled partially. Particularly the well designed AMOS study [23-27] enrolled a quite heterogeneous cohort of patients – albeit this may reflect the "real life" situation in most medical wards.

The criteria for selection of patients in the study of Majorek et al. [10] are unclear. In the study of Fischer and Rheingans [28], which described the results quite well, one can not ignore potential bias problems, and poorly defined primary aims; moreover, because all patients were in a health resort for several months, one may expect a spontaneous improvement of the clinical situation anyway.

The exploratory pre-post study of Bräuner-Gülow and Gülow [29] had a good description of the EYT related diagnostic criteria and identified types, and the EYT effects, but lacks a control group.

In most studies, EYT was used as an add-on, not as a mono-therapy. Just one study [10] explicitly investigated the effects of EYT in 5 ADHD boys, which did not receive additional medication (just one with additional methylphenidate medication). The evaluations of Hamre's group [23-27] described the outcome effects of a "therapy package", which included EYT and other medications.

### Therapeutic Effects

**Figure 1:**
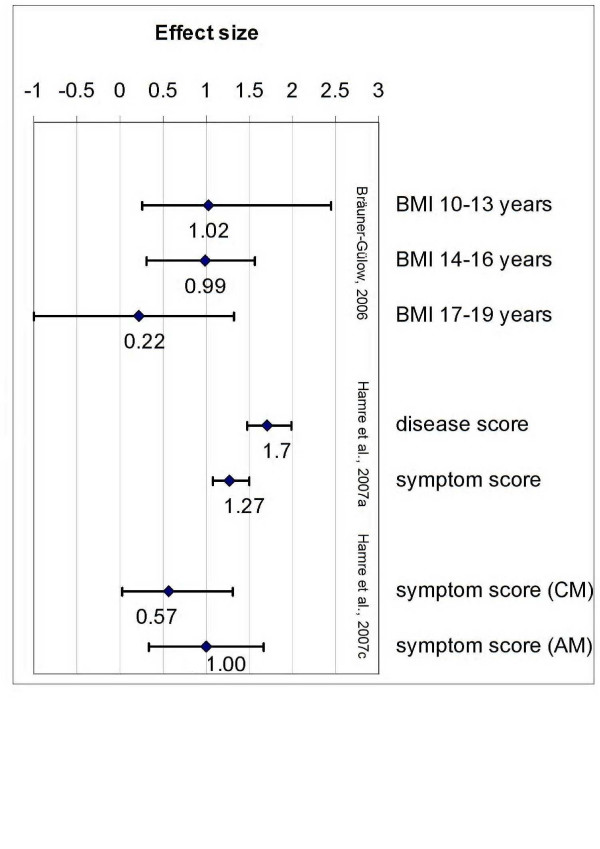
**Effect sizes (Cohen's d) of studies on EYT**. Analysed variables of the respective studies were indicated. Effect sizes were classified as small (0.20–0.49), medium (0.50–0.79), and large (≥ 0.80).

First we analyzed the prospective AMOS cohort study [23,25,26] with a pre-post design but without a control group (Tables 2 and 3). This study was a 4-year prospective cohort study and referred to patients with various chronic diseases such as mental, respiratory, musculoskeletal, headache etc. One evaluation [25] specifically addressed the effects of EYT which was used in 94%, adjunctive AM medication in 75%, and other treatments (but 63% of patients were without diagnosis-related adjunctive therapy). As shown in Table 3 and Figure 1, the authors reported significant improvement of disease and symptom scores (effect sizes for 0–12 months comparison: 1.34 and 1.04; Cohens'd indicate large effects with respect to both variables) and quality of life (SF-36 Health Change for Adults, and KITA Psychosoma and Daily Life subscales for children: 0.41–0.67), but also a slight increase of psychotherapy usage. All other published clinical outcome details of the AMOS study were summarized in Tables 2 and 3.

**Table 3:** Clinical studies enrolling therapeutic eurythmy (with and without add-on treatments)

**Ref**.	**Treatment**	**Results**	**Effect size (Cohen's d)**
**[28]**	EYT; additionally: CO_2 _bathes, swimming, massages, inhalations, diet, health related lectures	Physiological and psychomental reactions similar to control group with active training, particularly psychomental strain and others better in the EYT group	/
**[10]**	EYT, 30 min. once a week (7 – 25 sessions per child); no other therapy during study, but 1 child with methylphenidate medication	Positive changes (normalisation) of movement skills (5/5) and concentration (4/5); minor improvement on working speed (5/5); slight improvement of social behaviour problems (4/5) and slight drop of hyperactivity (4/5); stopping of methylphenidate medication at the end of the study in 1/1	/
**[29]**	EYT, 25 min. 3–5x per week, 6–14 weeks treatment period during hospital stay; additional treatments: psychotherapy, change of eating habits, art therapy, patient caring procedures	Improvement of Body Mass Index in 10–13 year old girls (pre-post Standardised Response Mean: 0.79), in 14–16 year old girls (pre-post Standardised Response Mean: 1.08), but not in 17–19 year old girls (pre-post Standardised Response Mean: 0.23); improvement of cold of limbs (55/67) and constipation (38/54); artificial feeding not required any longer (0/54); no changes in amenorrhea (53/53); improvement of fluent movements, decrease of movement compulsion etc.; improvement of body perception	10–13 years: 1.02 (0.26–2.45; large effect); 14–16 years: 0.99 (0.31–1.57; large effect); 17–19 years: 0.22 (-1.00–1.32; small effect)
[23]	EYT (47%), AM art therapy (18%), rhythmic massage (10%), AM medication (26%); median therapy duration 120 days (interquartile range 81–195); median number of therapy sessions 12 (interquartile range 10–20)	Significant improvement of disease and symptom scores (Standardised Response Mean for 0–6 months: 1.23 and 1.09), and quality of life (SF-36, KINDL and KITA)	
[24]	AM medication (13%) and AM therapies (87%), such as art therapy (43%), EYT (37%), rhythmical massage therapy (6%); median therapy duration 137 days (interquartile range 91–212); 29% used antidepressants within the first 6 study months, 24% had at least 10 psychotherapy sessions, 55% had no standard therapy (i.e. psychotherapy, antidepressants)	Significant improvement of depression (CES-D: Standardised Response Mean for 0–12 months: 1.20 [art therapy 1.32, painting, drawing, clawing 1.25, EYT 1.08]), disease and symptom scores (1.77 and 0.91), and quality of life (SF-36 mental: 1.11)	
**[25]**	**EYT (94%) **with a median therapy duration 119 days (interquartile range 84–188), median number of therapy sessions 12 (interquartile range 10–19); adjunctive AM medication in 75%; other treatments were used too (but 63% of patients were without diagnosis-related adjunctive therapy)	Significant improvement of disease and symptom scores (Standardised Response Mean for 0–12 months comparison: 1.34 and 1.04) and quality of life (SF-36 and KITA: 0.41–0.67);; significant increase of psychotherapy usage	disease score: 1.70 (1.47 – 1.99; large effect); symptom score: 1.27 (1.08–1.50; large effect)
[26]	AM medication (71–86%), AM art therapy (4%) and EYT (14%); other treatments were used too (but 63% of patients were without diagnosis-related adjunctive therapy)	Significant improvement of disease and symptom scores (Standardised Response Mean for 0–12 months comparison: 1.52 and 1.04, and quality of life (SF-36 and KITA: 0.33–0.71); Significant increase of AM medication and psychotherapy usage	
**[27]**	AM therapy (incl. 70% EYT, 24% rhythmical massage, 6% art) versus conventional therapies	Significant improvement of symptom scores (pre-post Standardised Response Mean for 0–12 months comparison: 1.0 in AM vs. 0.5 in control) and physical health score (SF-36 Standardised Response Mean: -0.8 in AM vs. -0.2 in control) in both groups; after adjustment, in AM group more pronounced improvements for mental health, general health and vitality	symptom score conventional group: 0.57 (0.02 – 1.31; moderate effect), AM group: 1.00 (0.33–1.67; large effect)

It is worth to mention that adverse reactions to EYT occurred just in 3% (3/419) of patients, but none stopped EYT due to the adverse reactions [25]. The authors mentioned the absence of a comparison group receiving another treatment or no therapy, but countered that "non-AM adjunctive therapies cannot explain the improvement of Disease and Symptom scores, since the improvements were even more pronounced in patients nut using such therapies".

Next we analyzed the two exploratory pre-post studies without a control group (Tables 2 and 3). The study by Bräuner-Gülow and Gülow [29] investigated 70 girls respectively women with Anorexia nervosa, which received EYT, psychotherapy, change of eating habits, art therapy, and patient caring procedures. None of them required artificial feeding at the end of the hospital stay. The authors reported an improvement of cold of limbs (55/67) and constipation (38/54), but no changes in amenorrhea. Moreover, the therapists reported improvement of fluent movements, decrease of movement compulsion etc.; and improvement of body perception. The body mass index of the girls improved, particularly in the younger girls: effect size 10–13 year old girls pre-post: 0.79, and in 14–16 year old girls 1.08, but not in 17–19 year old girls (effect sizes pre-post: 0.23). Cohen's d indicate large effects for the younger girls, and small effects for the older girls (Table 3; Figure 1).

The exploratory pre-post case study without control group by Majorek et al. [10] investigated the effect of EYT in 5 boys with attention deficit hyperactive disorder (ADHD). Just one of them received additionally methylphenidate. The authors reported positive changes in movement skills and concentration, minor improvement on working speed, slight improvement of social behaviour problems and slight drop of hyperactivity (Table 3).

Two studies had a control group or a comparison design (Tables 2 and 3). The study with a prospective non-randomized comparison design which is part of the AMOS study [27] enrolled patients with chronic low back pain (Table 3). It revealed significant improvement of symptom scores (calculated effect sizes for 0–12 months comparison: 1.0 in AM vs. 0.5 in control; as shown in Table 3 and Figure 1, Cohen's d indicate large effects for AM group and moderate effect for conventional group) and physical health (SF-36 effect size: -0.8 in AM vs. -0.2 in control) in both groups, while in the AM group more pronounced improvements for mental health, general health and vitality were observed. With 34 patients in the AM group (enrolled by 25 physicians), and 28 patients in the conventionally treated group (enrolled by 13 physicians), the sample size of the study was low.

The other study with a controlled design investigated 39 patients in a health resort and a complex therapeutic approach [28]. The authors measured blood pressure, physical capacity and several other physiological parameters. Although both groups were not recruited at the same time, the treatment effects were similar in both groups, EYT and physical training respectively. Several positive effects, particularly psycho-mental strains, were stronger in the EYT group. However, the statistical analyses were pre-post analyses of the respective group, and did not test between the groups. The overall results of the study reflect an effective stay at a health resort with intended normalization of blood pressure and other parameters.

## Discussion

Indications, study designs, outcome parameters and the usage of additional treatments within the few identified studies enrolling EYT were quite heterogeneous. Because non-pharmacological interventions in CAM such as EYT do not receive relevant financial support, well designed controlled studies are rare. Nevertheless, some of the studies reported here had a satisfactory quality and an appropriate data presentation. In contrast, the retrieved German language case-reports had several methodological flaws and obscurities, one can not exclude a positive publication bias, and thus they can not be regarded as a valid ground to approve the clinical effects of EYT. A possible bias factor which is difficult to exclude could be the specific AM background of the research groups and therapists; however, one may assume this bias in most articles on unique CAM remedies. Hamre et al. [25] and Kienle et al. [9] analysed several potential bias factors in EYT and AM and performed sensitivity analyses, which all indicated validity of published data.

Nevertheless, the retrieved clinical studies describe significant improvements of the symptoms, which can be ascribed to effects of the multimodal treatment approaches of AM. In the study of Majorek et al. [10], one has to state impressive treatment effects of EYT, which was used as the sole therapy in 4/5 boys. In all other studies, one may assume that EYT at least has contributed significantly to the positive effects – without any relevant side effects. It is difficult to judge the unique contributions of the different AM therapies used as a "therapy package". From a therapeutic point of view this is a comprehensible necessity to address different "aspects" of a patient, but from an analytical point of view one would rather investigate the effects of the constituents of this "therapy package".

In fact, Hamre and co-workers stated that AM therapies "were evaluated as a therapy package, the question of specific therapy effects vs. non-specific effects (placebo effects, context effects, patient expectations etc.) was not an issue of the present analysis" [24]. Although in the prospective cohort studies of Hamre et al. [24,26], the impact of EYT was somewhat lower than that of other AM therapies, they were nevertheless effective – and addressed other relevant aspects of the patients, which contributed to a process of healthy improvement.

From a theoretical point of view there is a sound rationale to apply EYT in patients [11,30], while the empirical evidence for its essential use is unclear. The most common diagnoses in Hamre's studies ranged from "severe" to "moderate", i.e. malignancies, mental/mood disorders, neck-shoulder-arm pain, back pain, headache/migraine, asthma, sinusitis, fatigue, and other diagnoses [23-26]. Although one may assume that some of these diseases may improve in response towards a therapeutic intervention and during time anyway, more than 95% of these patients had median disease duration of 3 years (IQR 0.5 – 9.8 and 1.0 – 8.5, respectively), and thus a suggested "spontaneous recovery" of symptoms with the onset of AM interventions in all cases is implausible. Nevertheless, without disease related cohorts or a control group, which is lacking in most of the studies, it is difficult to substantiate the assumed effectiveness of the therapeutic strategies, particularly EYT. However, Hamre et al. [25] performed two bias analyses, first a dropout bias analysis (which reducing the average 0–12 months improvements by 19% and 4%, respectively), and the second addressing the effects of relevant adjunctive therapies. It was mentioned that restricting the sample to patients not using diagnosis-related adjunctive therapies during the first six study months, the average Disease and Symptom Score improvements were increased by 10% and 6%, respectively [25]. Albeit it is a single-arm therapy study with all it's limitations, the study enrolled a large number of patients, had a high follow up period and high follow up rates, and can be regarded as a qualitatively well performed study which addressed several other relevant bias factors [9,25].

Taken together, one may suggest that EYT could be an relevant contribution of AM in the treatment of diseases particularly with (psycho-)neurological movement affections [31]. This unique intervention might be an interesting treatment option in conventional health care system, and thus should be investigated also in a non-anthroposophical context, but with trained therapists. For the patients, there is no need to agree with the views of AM, but they are advised to follow the external movements of EYT with guided imagery. A recent study on the physiological effects of EYT showed that EYT with internal "movement" was performed much slower than the sole movement without guided imaginary, and thus effects on heart rate variability were more pronounced in the first group (Cysarz et al., in preparation).

## Conclusion

Although indications, study designs, outcome parameters, and the usage of additional treatments within the identified studies enrolling EYT were quite heterogeneous, these studies describe improvements of the symptoms (with relevant effect sizes in most cases; Figure 1), which can be ascribed to effects of the multimodal treatment approaches of AM which enrol EYT. EYT could be regarded as a potentially relevant add-on in a complex therapeutic concept which intends to support health and well-being (salutogenesis), although its specific relevance remains to be clarified. Well performed controlled studies with defined indications and treatment regimes are highly recommended.

## List of abbreviations

AM – anthroposophic medicine; CAM – complementary or alternative medicine; EYT – eurythmy therapy

## Competing interests

PFM, AB, and TO received financial support with a grant of the Raphael Medical Centre, Hildenborough, Kent, GB, and a grant by the Software AG Stiftung, Darmstadt, Germany. MM has no competing interests.

## Authors' contributions

AB, TO and MM contributed to data collection. AB and TO analyzed and reviewed the data. PFM contributed to draft the manuscript. AB was principle author of the paper, and had full access to the data. All authors contributed to manuscript revision and approved the final manuscript.

## Pre-publication history

The pre-publication history for this paper can be accessed here:



## References

[B1] Bausell R, Lee WL (2001). Demographic and health-related correlates of visits to complementary and alternative medical providers. Med Care.

[B2] Linde K, Streng A, Hoppe A, Weidenhammer W, Wagenpfeil S, Melchart D (2007). Randomized trial vs. observational study of acupuncture for migraine found that patient characteristics differed but outcomes were similar. J Clin Epidemiol.

[B3] Linde K, Witt CM, Streng A, Weidenhammer W, Wagenpfeil S, Brinkhaus B, Willich SN, Melchart D (2007). The impact of patient expectations on outcomes in four randomized controlled trials of acupuncture in patients with chronic pain. Pain.

[B4] Steiner R, Wegman I (2000). Extending practical medicine Fundamental principles based on the science of the spirit (GA 27).

[B5] Ritchie J, Wilkinson J, Gantley M, Feder G, Carter Y, Formby J (2001). A model of integrated primary care: anthroposophic medicine.

[B6] Büssing A (2000). Mistletoe The Genus Viscum Medicinal and Aromatic Plants – Industrial Profiles.

[B7] Kienle GS, Kiene H (2003). Die Mistel in der Onkologie Fakten und konzeptionelle Grundlagen.

[B8] Kienle GS, Berrino F, Büssing A, Portalupi E, Rosenzweig S, Kiene H (2003). Mistletoe in Cancer. A Systematic Review on Controlled Clinical Trials. Eur J Med Res.

[B9] Kienle GS, Kiene H, Albonico HU (2006). Anthroposophische Medizin in der klinischen Forschung Wirksamkeit, Nutzen, Wirtschaftlichkeit, Sicherheit.

[B10] Majorek M, Tuchelmann T, Heusser P (2004). Therapeutic Eurythmy-movement therapy for children with attention deficit hyperactivity disorder (ADHD): a pilot study. Complement Ther Nurs Midwifery.

[B11] von Laue HB, von Laue EE (2007). Zur Physiologie der Heileurythmie.

[B12] Stroup DF, Berlin JA, Morton SC, Olkin I, Williamson GD, Rennie D, Moher D, Becker BJ, Sipe TA, Thacker SB (2000). Meta-analysis of observational studies in epidemiology: a proposal for reporting. Meta-analysis Of Observational Studies in Epidemiology (MOOSE) group. JAMA.

[B13] Cohen J (1988). Statistical power analysis for the behavioral sciences.

[B14] Wolf FM (1986). Meta-analysis: Quantitative Methods for Research Synthesis.

[B15] Ernst E (2004). Eurythmie beruhigt den Zappelphilipp. MMW Fortschr Med.

[B16] Hamre HJ, Witt CM, Glockmann A, Ziegler R, Willich SN, Kiene H (2006). Health costs in anthroposophic therapy users: a two-year prospective cohort study. BMC Health Serv Res.

[B17] Heusser P, Braun SB, Ziegler R, Bertschy M, Helwig S, van Wegberg B, Cerny T (2006). Palliative in-patient cancer treatment in an anthroposophic hospital: I. Treatment patterns and compliance with anthroposophic medicine. Forsch Komplementarmed.

[B18] Heusser P, Braun SB, Bertschy M, Burkhard R, Ziegler R, Helwig S, van Wegberg B, Cerny T (2006). Palliative in-patient cancer treatment in an anthroposophic hospital: II. Quality of life during and after stationary treatment, and subjective treatment benefits. Forsch Komplementarmed.

[B19] Gödl R, Glied N, Muhry F, Früwirth M, Messerschmidt D, Niederl T, Rissmann W, Lehofer M, Moser M, Heusser P (1999). Überwärmungsbäder bei depressiver Erkrankung – Veränderung der vegetativen Balance. Akademische Forschung in der Anthroposophischen Medizin Beispiel Hygiogenese: Natur- und geisteswissenschaftliche Zugänge zur Selbstheilungskraft des Menschen.

[B20] Schaper LC (1996). Effekte einer wiederholten Hyperthermiebehandlung durch Überwärmungsbäder auf die Produktion von Interleukin-6 sowie auf die mittlere Körpertemperatur und den psychopathologischen Befund bei Patienten mit depressiven Störungen.

[B21] Simon L, Schietzel T, Gärtner C, Kümmell HC, Schulte M (1997). Ein anthroposophisches Therapiekonzept für entzündlich-rheumatische Erkrankungen. Ergebnisse einer zweijährigen Pilotstudie. Forsch Komplementarmed.

[B22] Martini I (1999). Anwendungsbeobachtungen mit Iscucin-Quercus bei ehemals i.v.-Drogenabhängigen mit chronischer Hepatitis C des Genotyp 1 in SYNANON Schmerwitz. Der Merkurstab.

[B23] Hamre HJ, Becker-Witt C, Glockmann A, Ziegler R, Willich SN, Kiene H (2004). Anthroposophic therapies in chronic disease: the Anthroposophic Medicine Outcomes Study (AMOS). Eur J Med Res.

[B24] Hamre HJ, Witt CM, Glockmann A, Ziegler R, Willich SN, Kiene H (2006). Anthroposophic therapy for chronic depression: a four-year prospective cohort study. BMC Psychiatry.

[B25] Hamre HJ, Witt CM, Glockmann A, Ziegler R, Willich SN, Kiene H (2007). Eurythmy therapy in chronic disease: a four-year prospective cohort study. BMC Public Health.

[B26] Hamre HJ, Witt CM, Glockmann A, Ziegler R, Willich SN, Kiene H (2007). Anthroposophic medical therapy in chronic disease: a four-year prospective cohort study. BMC Complement Altern Med.

[B27] Hamre HJ, Witt CM, Glockmann A, Wegschnieder K, Ziegler R, Willich SN, Kiene H (2007). Anthroposophic vs. conventional therapy for chronic low back pain: a prospective comparative study. Eur J Med Res.

[B28] Fischer K, Rheingans H (1985). Vergleichende Untersuchung einer künstlerisch-übenden mit einer konventionell aktiv-trainierenden Kurbehandlung an Herz- und Kreislaufkranken mit einer Herzinfarktgruppe. Erfahrungsheilkunde.

[B29] Bräuner-Gülow G (2006). Heileurythmie bei Magersucht im Jugendalter. Methodik zur Bewegungsanalyse. Aspekte zur Diagnostik, Bewegungstherapie und Forschungsstand. Zusammenfassung einer Pilotstudie von 2002–2005. Der Merkurstab.

[B30] Edelhäuser F, Steinke U (1998). Zur Einführung. Lesebuch Heileurythmie Aufzeichnungen aus langjähriger Praxis für Patienten, Ärzte, Therapeuten.

[B31] Steinke U (1998). Lesebuch Heileurythmie Aufzeichnungen aus langjähriger Praxis für Patienten, Ärzte, Therapeuten.

